# Group velocity mismatch-absent nonlinear frequency conversions for mid-infrared femtosecond pulses generation

**DOI:** 10.1038/srep10887

**Published:** 2015-06-23

**Authors:** Haizhe Zhong, Lifu Zhang, Ying Li, Dianyuan Fan

**Affiliations:** 1 SZU-NUS Collaborative Innovation Center for Optoelectronic Science & Technology, Key Laboratory of Optoelectronic Devices and Systems of Ministry of Education and Guangdong Province, College of Optoelectronic Engineering, Shenzhen University, Shenzhen 518060, China

## Abstract

**A novel group velocity mismatch (GVM) absent scheme for nonlinear optical parametric procedure in mid-infrared was developed with type-I quasi phase matching by use of an off-digital nonlinear optical coefficient** ***d***_**31**_**. This was achieved by matching of the group velocities of the pump and the signal waves, while the phase velocities were quasi phase matched. The system employs MgO-doped periodically poled LiNbO**_**3**_ **as the nonlinear medium. Desired group-velocity dispersion would be obtained via appropriately temperature regulation. To demonstrate its potential applications in ultrafast mid-infrared pulses generation, aiming at a typical mid-infrared wavelength of ~3.2 μm, design examples of two basic nonlinear frequency conversion procedures are studied for both the narrow-band seeding mid-IR optical parametric amplification (OPA) and the synchronously pumped femtosecond optical parametric oscillation (SPOPO). Compared with the conventional scheme of type-0 QPM, the quantum-efficiency can be more than doubled with nearly unlimited bandwidth. The proposed GVM- absent phase matching design may provide a promising route to efficient and broadband sub-100 fs mid-infrared ultrafast pulses generation without group-velocity walk-off.**

The mid-infrared (mid-IR) spectral region of 2–20 μm is attractive in many areas of science and technology. The mid-IR region contains the important transparent window of the Earth’s atmosphere (3–5 μm) and nearly all fundamental rovibrational absorption bands of molecules^1^,^2^. Ultrashort-pulsed light sources in this region are sought for numerous different fields ranging from fundamental strong field physics to medical and industrial applications^3^,^4^,^5^. As the application area expands, it is urgently thirsty for high intensity mid-IR femtosecond laser sources of broad spectrum, high time-resolution, and wavelength tunable.

On the other hand, due to the limitation of suitable laser gain media, practical femtosecond laser sources are mostly limited to Ti:sapphire and Er:fiber lasers of less than 2 μm, and remained scarce at wavelengths beyond 3 μm. In most cases, to access this notorious spectral region, nonlinear optical effects are harnessed to transfer electromagnetic energy from the visible or near-infrared (near-IR) domain into the mid-IR. So far, high-repetition-rate mid-IR femtosecond pulses at wavelengths exceeding 3 μm have been produced mainly by means of the difference-frequency generation (DFG) between two temporally synchronized pulses and the synchronously pumped optical parametric oscillator (SPOPO)^6^,^7^,^8^,^9^.

However, to expand the previously developed picosecond frequency conversion technology to femtosecond, the effects of group-velocity mismatch (GVM) begin to play a substantial role for shorter pump pulses, which makes the frequency transformation of broadband femtosecond sources from near-IR to mid-IR faces predominant challenges^10^,^11^,^12^. In the view of temporal walk-off, as shown in Fig. 1(a), the pump and signal waves would walk away from each other during the interaction, because of their different speeds in the material medium. Intuitively, this effect prevents the mid-IR signal from lasting obtaining energy from the near-IR pump pulse due to their temporal un-overlap, and the infrared radiation will be generated over a wide temporal range. In the spectrum domain, GVM determines the phase matching (PM) bandwidth of parametric process. A large GVM would dramatically decrease the spectrum bandwidth and thus extend the transform-limited pulse duration of generated mid-IR pulses. In some sense, group velocity matching and broadband PM are equivalent concepts, and the idea of broadband optical frequency conversion is equivalent to the group-velocity matching between the interacting waves. At present, high-intensity femtosecond lasers are still limited in near-IR, such as the Ti: sapphire at 800 nm. Due to the considerably distinct between the near- and mid-IR wavelengths, the effects of GVM would be much more detrimental for few-cycle mid-IR pulses generation.

The most direct way to alleviate the GVM effects and broaden the PM bandwidth is to reduce the interaction length. However, the efficiency of frequency conversion is inversely proportional to the square of the interaction length, leading to a poor trade-off between the bandwidth and the conversion efficiency. Employing a non-collinear geometry can allow a broad PM bandwidth at the cost of introducing hardly offset idler angular dispersion^13^. Even so, there is no guarantee that this technology can be employed in any nonlinear interaction. Unfortunately, parametric amplification in mid-IR with typical near-IR pump sources is not one of the feasible cases^14^. Let the optical nonlinear interaction operate at nearly degeneracy may inherently match the signal and idler group velocities^15^. Whereas, subject to the limited pump sources, this scheme can be realized only at few signal wavelengths. On the other hand, a more sophisticated method based on aperiodically poled lithium niobate (APPLN) was proposed^16^,^17^,^18^. In these domain-engineered Quasi-Phase-Matching (QPM) structures, PM condition can be satisfied at different positions for distinct spectra components. Nonetheless, this method amounts to a shorter the effective crystal length, and sets real obstacles for the design and fabrication of these crystals.

Aiming at the significant GVM issue in mid-IR frequency conversions because of the highly non-degenerate wavelengths, in this paper, we propose a novel QPM design capable of group-velocity matching between the near- and mid-IR waves. Periodically poled LiNbO_3_ (PPLN) is a representative QPM device that demonstrated impressive performance in optical frequency conversion because of its really large nonlinear coefficient, *d*_33_^19^,^20^. However, to make use of that nonlinear coefficient, satisfying the type-0 QPM condition is necessary, i.e., all of the pump, signal and idler waves should be e-polarized during the nonlinear process (Fig. 1(a)). In these cases, the QPM wavelength bandwidth would be extremely narrow because of its steep dispersion, limiting its broadband applications to ultrafast pulses^21^ Different from the traditional type-0 QPM interaction using *d*_33_, the proposed scheme plans to adopt the QPM technology which meets the type-I PM condition (o+o-›e) (Fig. 1(c)). Via appropriate temperature control of the periodically poled crystal, the GVM between the near-IR pump and mid-IR signal waves can completely vanish. On this basis, phase velocities are quasi phase matched at that operation temperature, resulting in a GVM-absent PM with extremely broad PM bandwidth comparable to the degenerate cases. In this paper, we start from the theoretical analysis of the dispersion behavior in MgO:PPLN at various wavelengths, which clearly shows that the velocity of e-polarized near-IR pulses at 800 nm can be matched with that of the o-polarized ones at mid-IR wavelengths of ~1.8–3.5 μm in a temperature range of 20–220 ^o^C. To demonstrate its potential applications on sub-100 fs ultrafast mid-IR pulses generation, we numerically study this novel frequency conversion scheme at the typical mid-IR wavelength of ~3 μm, through two basic nonlinear procedures, i.e., the narrow-band seeding OPA and synchronously pumped femtosecond OPO, which have been widely employed to produce mid-IR pulses. Temporal and spectral characterizations of the generated mid-IR pulses were carried out to visualize the effects of the proposed GVM-absent PM frame. Compared with the conventional counterparts used type-0 QPM, the quantum-efficiency can be more than doubled with nearly unlimited gain bandwidth. The GVM-absent PM design may provide a promising route to efficient and broadband sub-100 fs mid-IR ultrafast pulses generation without group-velocity walk-off. Additionally, for the proposed design, the grating period of MgO:PPLN would be significantly longer than the conventional cases (>50 μm), which is advantageous to the fabrication of periodically poled crystals with larger aperture and subsequently sustains higher pulse energy.

## Results

### Temperature-dependent group velocity in MgO:PPLN

For conventional nonlinear crystals based on angle-tuning PM, when group velocities are matched, the phase velocity matching condition usually cannot be satisfied simultaneously, resulting in poor conversion efficiency. In the QPM structure, the sign of the nonlinear coefficient is reversed just at the depth where the generated waves would start to oscillate out of phase. It ensures that any frequency conversion can be noncritically phase matched within the transparent wavelength range only if an appropriate poling period is chosen^22^. The key idea of the proposed GVM-absent PM is modifying the group-velocities of the interacting waves through temperature regulation. On this basis, phase velocities matching can be achieved simultaneously via QPM with a proper grating period. As we all know, refractive index is temperature-dependent, the group velocity should be temperature-relative as well. In Fig. 2 we plot the group velocity of ν_g_ for certain typical wavelengths versus the crystal temperature in both o and e polarization. The given *ν*_g_ was calculated based on the temperature-dependent Sellmeier equations of 5% doped MgO:PPLN, provided by the HCP company^23^. As shown by the calculated results, for the typical near-IR wavelength of 800 nm and mid-IR wavelengths of 1.8–3.4 μm, there is definitely remarkable difference between their group velocities when they are both in e-polarization, which seems to be indelible just via simply regulating the crystal temperature (Fig. 2(a)). In other words, even adding the degree of freedom of temperature-tuning, it is still impossible to set up a GVM-absent frequency conversion system under the conventional type-0 QPM condition. Whereas, when these waves transmit in the MgO:PPLN with orthogonal polarizations, i.e., 800 nm is e-polarized while 1.8–3.4 μm is o-polarized, their group velocities may be matched at certain temperatures, e.g., T = ~175 ^o^C for 800 nm and 3.2 μm (Fig. 2(b)). As a reference, the GVM between these waves is expected to be ~200 fs/mm for conventional type-0 interaction in the same MgO:PPLN. It should be noted that, to suppress the photorefractive damage, in general, MgO:PPLN should be heated and operate at temperatures above 100 °C. As shown in Fig. 2(b), supposing a typical 800 nm Ti:sapphire femtosecond laser serve as the pump source, the proposed GVM-absent scheme can be applied to generate ultrashort pulses at **~**1.8 to 3.4 μm in a temperature range of 20–220 ^o^C. In principle, this scheme can be adopted in much broader spectral region based on other pump sources, e.g., the familiar wavelength between 950 nm to 1050 nm.

**Figure 1:**
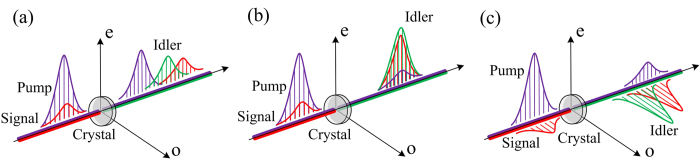
The role of group-velocity mismatch. Avoiding group-velocity mismatch is critical for ultrafast nonlinear interaction to obtain efficient conversion efficiency. (**a**) The widely used QPM scheme based on MgO:PPLN, in these cases, all of the interaction waves are e-polarized and significant GVM exists between the near-IR pump and mid-IR signal waves. (**b**) The ideal situation when interaction waves have unique group-velocity; (**c**) The suboptimal preference that pump and signal waves are group-velocity matched based on the proposed type-I QPM frame.

It is worth to note that QPM can also be established when the pump and signal waves travel with orthogonal polarizations. To eliminate the GVM influence, applying the type-I QPM technology plus with temperature regulation will be an appropriate choice which can solve the notorious problem essentially (Fig. 1(c)). Because of the uniform group velocity, in contrast to the conventional type-0 QPM interaction, theoretically, a considerable long nonlinear crystal can be used even for the ultrashort pulses interaction without concerning about the temporal walk-off between the pump and signal waves. More importantly, extremely broad PM bandwidth can be allowed for those optical frequency conversions, which is crucial to the generation of ultrafast mid-IR femtosecond laser pulses. Additionally, for the proposed design, the grating period of MgO:PPLN would be significantly longer than the conventional cases (>50 μm), which is advantage for the fabrication of periodically poled crystals with larger aperture and subsequently sustains higher pulse energy.

To verify the potential applications of the proposed GVM-absent PM scheme on ultrafast mid-IR pulses generation, we numerically study two basic frequency conversion processes commonly used to generate femtosecond mid-IR pulses, i.e., the narrow-band seeding OPA^24^,^25^ and synchronously pumped femtosecond OPO^26^,^27^,^28^, which have been widely employed to produce mid-IR pulses. Table 1 published the simulation parameters including the effective nonlinear coefficient of *d*_eff_, and the relevant material dispersion parameters of MgO:PPLN^29^,^30^. Besides the proposed GVM-absent design, the traditional type-0 QPM is also investigated and compared.

### Characteristics of quasi-cw-seeding OPA in mid-IR

**Figure 2:**
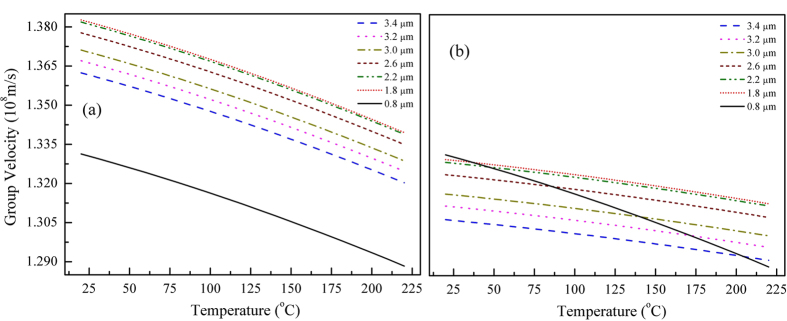
Group velocities for 800 nm (e-polarization) and a series of mid-IR wavelengths at 1.8–3.4 μm (both e-polarization (**a**) and o-polarization (**b**)) in 5-mol % MgO-doped LiNbO3 as a function of crystal temperature. All the data were calculated through temperature-dependent Sellmeier equations provided by the HCP Company^19^.

First of all, we begin with the simpler procedure of quasi-cw-seeding OPA which was commonly employed to generate tunable mid-IR femtosecond pulses. In the following numerical simulations, we assumed a commercial femtosecond Ti:sapphire regenerative amplifier near 800 nm to be employed as the pump source, while the signal is quasi-cw pulses derived from the Q-switched Nd:YLF laser used to pump synchronously the regenerative amplifier. In these cases, the wanted mid-IR laser pulses of ~3.2 μm will be generated simultaneously as the idler wave. To shorten the interaction length over which parametric amplification takes place and minimize the influence of material dispersion, the pump intensity was fixed at ~100 GW/cm^2^, slightly lower than the optical damage threshold of MgO:PPLN. The seeding intensity of narrowband signal pulses was set to be ~50 kW/cm^2^ with submicrosecond pulse duration. As shown in Fig. 2(b), in order to eliminate the GVM between ~0.8 μm pump and ~3.2 μm idler waves, MgO:PPLN should be heated to ~175 ^o^C, with a QPM period of ~62.8 μm.

**Figure 3:**
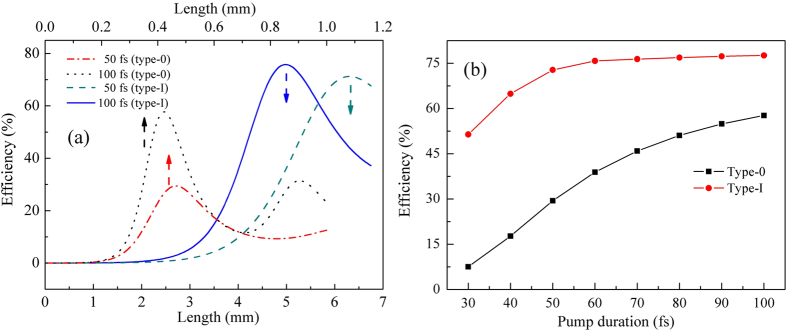
(**a**) Evolution of the quantum conversion efficiency with crystal length of MgO:PPLN, for both of the type-I and type-0 QPM designs. To show the plots clearly, different length scales were utilized for distinct QPM conditions. (**b**) The calculated maximum conversion efficiency versus various pump durations.

The quantum conversion efficiency with a dependence of crystal length was presented by numerically solving the nonlinear coupled-wave equations (see Methods for details), for both the type-I and type-0 QPM situations. As illustrated in Fig. 3(a), two specific examples are given, in which the pump duration is 50 fs and 100 fs respectively. To show the plots clearly, different length scales were utilized for distinct QPM situations. Taking the advantage of larger nonlinear coefficient, for the conventional type-0 QPM design, it requires a much shorter crystal length to achieve the limited conversion efficiency than the type-I cases. Whereas, whether the pump duration is 100 fs or just 50 fs, the maximum conversion efficiency type-I QPM can achieve were both markedly higher than those of their type-0 QPM counterparts (30%–100%). Figure. 3(b) presents the calculated maximum conversion efficiency versus various pump durations. As clearly shown in this figure, the shorter the pump durations, the more obvious the efficiency gap is, showing a more crucial influence of GVM during the nonlinear interaction. In comparison, the maximum conversion efficiency keeps relatively steady for the proposed scheme. Effective interaction length is quantified by the pulse splitting length, which is defined as the propagation length after which the signal pulse separates from the pump pulse, and subsequently determines the efficiency of nonlinear process. For one certain nonlinear process, the shorter the pulse duration, the influence of GVM would be more destructive ascribed to a shorter pulse splitting length. Because of the group velocity matching between the pump and signal waves, extremely broad PM bandwidth can be expected for the GVM-absent design.

**Figure 4:**
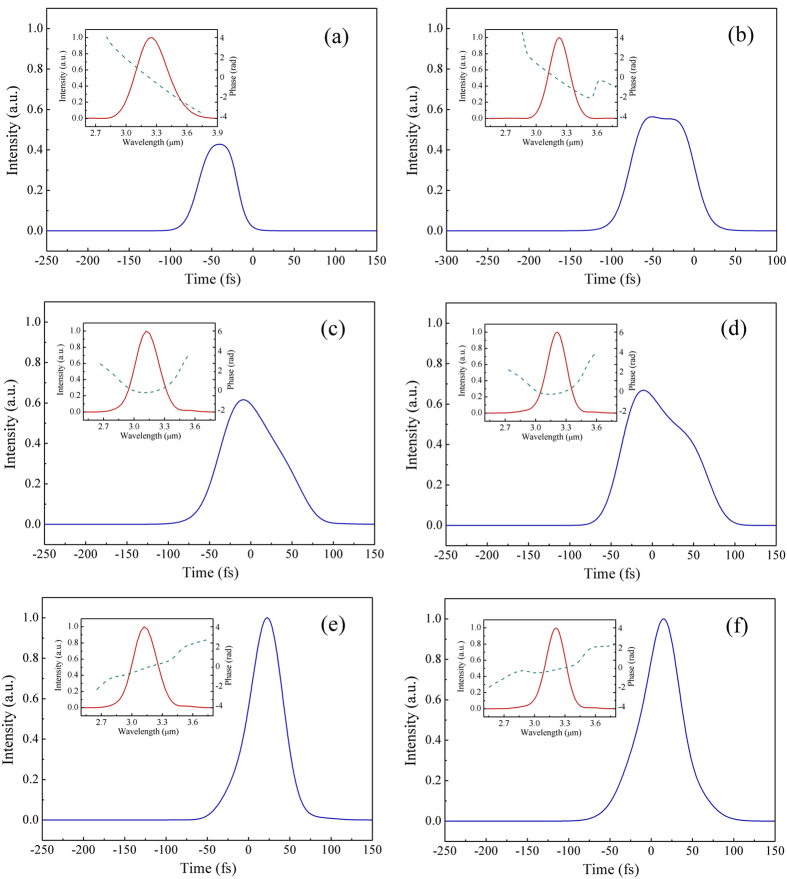
Simulated 3.2 μm idler waves of the narrow-band seeding OPA. The left column of (**a**) to (**e**) shows the temporal intensity of mid-IR pulses while the pump duration is 50 fs. From top to bottom, the type-0 (**a**) and type-I (**c**) QPM situations were considered, and the dispersion compensated pulses of type-I was presented in the bottom (**e**). The right hand column of (**b**) to (**f**) shows the cases of 100 fs pump duration. Inset: The corresponding spectral intensity and phase. The pulse intensity was respectively normalized to the compressed cases of (**e**) and (**f**) with the same pump duration.

For spectral and temporal characterization, the frequency spectrum and pulse envelope are given in Fig. 4 both types I and 0 respectively, when conversion efficiency reaches its maximum value. As we can see, for type-I QPM interaction (Fig. 4(c,d)), due to the longer interaction length caused by its relatively smaller effective nonlinear coefficient, the pulse envelope clearly suffered a significant influence of group-velocity dispersion, showing an obviously expanded pulse duration. Nonetheless, these pulses should be compressible by propagation in a properly chosen thickness of materials with positive dispersion in the mid-IR spectral region. The pulse envelopes went through dispersion compression were also presented in the bottom of Fig. 4. The nearly transform-limited mid-IR femtosecond pulses have perfectly preserved the temporal and spectral quality of the incident 800 nm pump pulses. In comparison, for their conventional type-0 QPM counterparts (Fig. 4(a,b)), the output pulse envelop appears slightly pulse distortion with much lower peak intensity (only ~50% of the optimum cases of (e) and (f)).

### Characteristics of mid-IR SPOPO

For the more complex frequency conversion procedure of SPOPO, we take a similar linear-cavity OPO model as that proposed by A. V. Smith *et al.*^31^. However, their approach assumes that all interaction waves have the same group velocity and is thus usually not appropriate for the picosecond or shorter pulses. In this paper, focus on the temporal property of OPO in femtosecond scale, our model makes some improvements and includes the group velocity mismatch and group velocity dispersion which play crucial roles in the femtosecond frequency conversions (see Methods for detailed descriptions).

**Figure 5:**
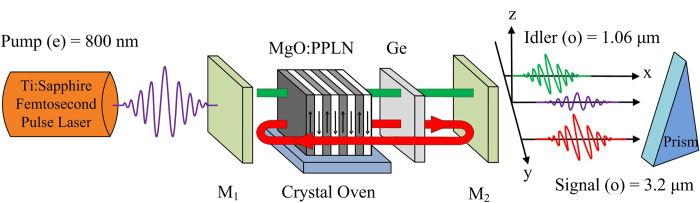
Schematic of the basic linear-cavity SPOPO setup. M_1_ is a dielectric mirror for in-coupling of the pump pulse; M_2_ is the out-coupler. The cavity length is matched to the repetition rate of pump laser.

A schematic of the basic SPOPO setup is depicted in Fig. 5. The SPOPO is designed with a basic linear cavity and singly resonant for the 3.2 μm mid-IR wavelength. A commercial Ti:sapphire femtosecond oscillator serves as the pump source, and the wavelengths of pump, signal, and idler are 0.8 μm, 3.2 μm and 1.06 μm, respectively. Through the in-coupling mirror of M_1_, pump pulses are coupled steered into the cavity. The nonlinear medium is a type-I QPM MgO:PPLN crystal with a poling period ∧ of ~62.8 μm. As previously discussed, because of the GVM-absent request, MgO:PPLN operates at ~175 °C in a temperature-control oven. Due to the singly resonant design, at the signal wavelength of 3.2 μm, the in-coupler is highly reflective, while the out-coupler is 80% reflective and 20% transmitted. In the meantime, M_2_ couples out both the residual pump and the by produced idler at 1.06 μm with high transmission in this spectral range. To achieve oscillation for the mid-IR signal wave, in addition to phase matching, the management of resonator’s dispersion is necessary. The broadband mid-IR pulses should be compressed to nearly transform-limited for each round trip. For this purpose, an additional plane-parallel plate of Ge (or ZnSe) having positive (1250 fs^2^/mm) GVD at 3.2 μm^32^ is inserted inside the cavity to compensate the negative GVD of MgO:PPLN (−760 fs^2^/mm).

**Figure 6:**
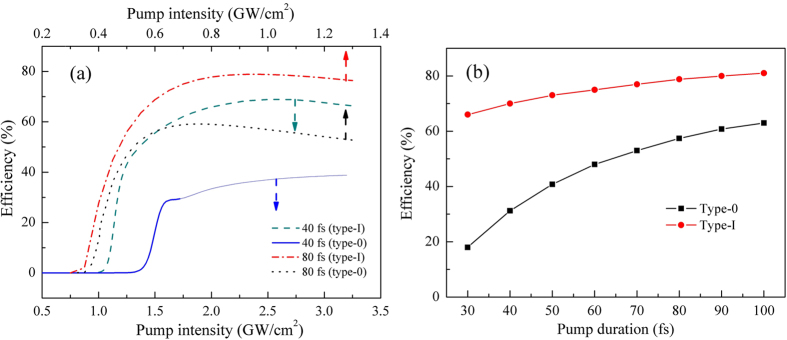
(**a**) Dependence of the photon conversion efficiency of SPOPO on pump intensities, for both type-I and type-0 QPM designs. To show the plots clearly, different pump intensity scales were utilized for distinct pump durations. The paler part in the solid curve correspond the cases when pulse distortion exists. (**b**) The calculated maximum conversion efficiency versus various pump durations without compromising on the quality of mid-IR output.

Figure 6 (a) presents the simulated dependence of quantum efficiency with different pump intensities. In this figure, two specific examples are given, where the pump duration is 80 fs and the shorter 40 fs respectively. For comparison, the widely used type-0 QPM is included as well. In the calculations, incident pump wave is taken to be Gaussian in temporal. Under the ideal situation of ignoring the influence of material dispersion, if we want to obtain same conversion efficiency for both type-I and type-0 QPM interactions, a shorter crystal length will be needed for the type-0 cases under the same pump intensity, due to its larger effective nonlinear coefficient of ~16 pm/V^33^. In our simulations, ~1 mm and ~5.5 mm MgO:PPLN were chosen for the type-0 and type-I QPM designs respectively, the ratio of which is just the inverse proportion of their nonlinear coefficients. Accordingly, ~0.5 and 2.7 mm-thick Ge plates were inserted inside the cavity to compensate the negative GVD of MgO:PPLN. In the circumstances, taking the detrimental GVD and GVM into account, the performance of different OPOs would be reflected in their conversion efficiencies and spectral bandwidths. As can be seen, for the proposed GVM-absent design with a 5.5 mm type-I QPM MgO:PPLN crystal, maximum photon efficiency of ~80% is obtained at the pump intensity of ~0.9 GW/cm^2^ while the pump duration is 80 fs. Comparatively, the maximum conversion efficiency would be less than 60% at ~0.7 GW/cm^2^ for the conventional type-0 QPM situation. It should be noted that, in the numerical simulations, the pulse slipping between the pump and signal waves had been compensated before the next pump pulse arrive, which can be realized by slightly modulating the cavity length in practice.

**Figure 7:**
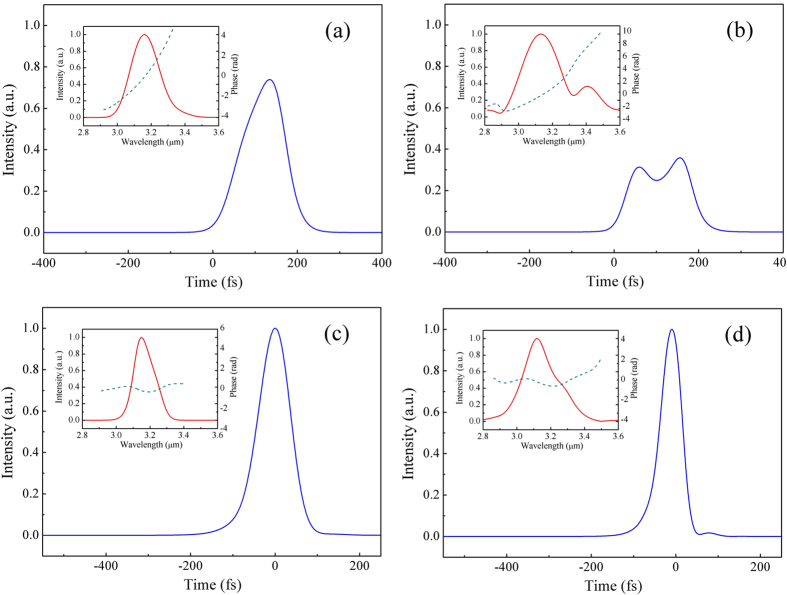
Simulated mid-IR outputs of the femtosecond SPOPO. The left column of (**a**) and (**c**) shows the temporal intensity while the pump duration is 80 fs. From top to bottom, the type-0 (**a**) and type-I (**c**) QPM situations were considered respectively. The right hand column of (**b**) and (**d**) shows the cases of 40 fs pump duration. Inset: The corresponding spectral intensity and phase. The pulse intensity was respectively normalized to the ones of type-I QPM design with the same pump duration.

For the shorter 40 fs pump pulses, abnormally, the conversion efficiency of type-0 QPM scheme increases steadily as the pump intensity arises. This weird phenomenon may be ascribed to the serious pulse distortion which starts at ~1.7 GW/cm^2^. The pulse envelope and corresponding frequency spectrum are given in Fig. 7(b), under a pump intensity of ~2.4 GW/cm^2^. One can clearly see that, due to the significant GVM and the shorter pulse duration, mid-IR signal pulses appeared as a severe distorted structure with two peaks. For the other cases, the frequency spectrum and pulse envelope for both types I and 0 QPM are also given, respectively, when the quantum efficiency is maximum. In comparison, nearly transform-limited clean pulses with similar temporal duration as the initial pump pulses were produced, under the same pump intensity for the proposed GVM-absent design. For a more general description, the calculated maximum conversion efficiency versus various pump duration is presented (Fig. 6(b)). All of the data was recorded before remarkable pulse distortion turns up. It shows that the efficiency gap would be more significant for the shorter pump duration. As the same of narrow-band seeding OPA discussed earlier, the proposed scheme shows a unique advantage in the ultrafast mid-IR pulses generation. The conversion efficiency can be more than doubled with nearly unlimited bandwidth when pump duration is less than 50 fs.

## Discussion

In this paper, we propose a novel QPM design capable of GVM-absent PM between the near- and mid-IR waves. To demonstrate its potential applications in sub-100 fs ultrafast mid-IR pulses generation, two basic nonlinear frequency conversion procedures of narrow-band seeding OPA and SPOPO were numerically studied at the typical mid-IR wavelength of ~3.2 μm. The simulation results have shown that, the quantum-efficiency can be more than doubled with nearly unlimited bandwidth compared with the conventional counterparts of type-0 QPM. Theoretically, for the nonlinear processes involving three individual interaction waves, no matter which PM scheme we employed, in general, only two of the traveling group velocities can be matched in the meantime. As future directions, for various frequency conversion processes, according to their different requirements on the relationship of group velocity between pump, signal and idler, we can employ the proposed PM technology to tailor the material dispersion through precisely temperature manage and satisfy the optimum situation. The demonstrated GVM-absent design may provide a promising route to achieve efficient and broadband ultrafast mid-IR pulses generation without group-velocity walk-off.

## Methods

### Nonlinear coupled-wave equations

Employing the slowly varying envelope and plane-wave approximation, the equations which govern the envelopes *A*_*s*_, *A*_*i*_ and *A*_*p*_ of signal (*s*), idler (*i*) and pump (*p*) lasers in the nonlinear interactions, respectively, are


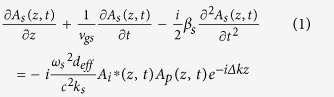



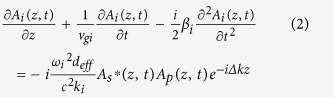



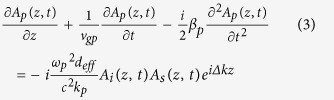


which have captured the main issues of GVM, group velocity dispersion (GVD), and strong energy exchange among the ultrafast parametric frequency conversions. In above equations, ν_g_ = ∂ω/∂*k* and *β* = ∂*k*/∂ω are the group velocity and the group velocity dispersion respectively. *n*, *ω* and *k* represent the refractive index, central frequency and wave vector. *d*_eff_ denotes the effective nonlinear coefficient, and ∆*k* = *k*_*p*_ − *k*_*i*_ − *k*_*s*_ is the phase mismatch among the interacting fields.

### Numerical model of mid-IR SPOPO

A conceptual description of the physical model of SPOPO is given below. All radiations of the three interaction waves interplay within the OPO cavity for many round trips. At each round trip, the initial pump and idler pulses start anew as the synchronous pump requires. The initial pump pulses propagate through the in-coupler and combine with the signal already in the cavity. After the nonlinear frequency conversion in MgO:PPLN, amplified signal pulses are again propagated back to the input coupler back and forth while a portion of energy transmit through the out-coupler as export. In the meantime, the residual pump wave and the byproduct of idler transmit out of the optical cavity in company. This process is repeated for each pump and signal pulse. Mathematically, these are represented by the following initial value boundary conditions^34^:


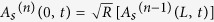



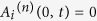



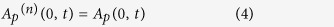


where *n* indicates the number of round trips, *R* represents the total signal reflectivity in the resonator, and *L* is the length of nonlinear crystal. A Gaussian profile is assumed for the initially incident pump pulse, *A*_*p*_(0,*t*) = *A*_0_ *exp*(−*t*^2^).

To simulate the evolution of these pulses in each round trip, the forward nonlinear coupled-wave equations of (1) to (3) are solved numerically by the standard split-step Fourier approach exploiting the fourth-order Runge-Kutta algorithm^35^, and the pump-to-signal energy conversion efficiency is evaluated by tempo-spatially integrating the intensity of the pulse.

## Additional Information

**How to cite this article**: Zhong, H. *et al.* Group velocity mismatch-absent nonlinear frequency conversions for mid-infrared femtosecond pulses generation. *Sci. Rep.* **5**, 10887; doi: 10.1038/srep10887 (2015).

## Figures and Tables

**Table 1:** Nonlinear Optical Crystal Parameters for 5% doped MgO:PPLN. (λ _pump_ = 800 nm, λ_mid_ = 3.2 μm, λ_near_ = 1.06 μm).

	*d*_eff_(pm/V)	GVM_pump-mid_(fs/mm)	GVM_pump-near_(fs/mm)	Temperature (^o^C)
Type-I	2.9	0	25.4	175
Type-0	16	−196	−174	21
	GVD_pump_ (fs^2^/mm)	GVD_mid_ (fs^2^/mm)	GVD_near_ (fs^2^/mm)	QPM period (μm)
Type-I	375	−760	278	62.8
Type-0	360	−636	233	22
